# Comparison of Clinical Characteristic and Prognosis between Ovarian Clear Cell Carcinoma and Serous Carcinoma: A 10-Year Cohort Study of Chinese Patients

**DOI:** 10.1371/journal.pone.0133498

**Published:** 2015-07-17

**Authors:** Shuang Ye, Jiaxin Yang, Yan You, Dongyan Cao, Huifang Huang, Ming Wu, Jie Chen, Jinghe Lang, Keng Shen

**Affiliations:** 1 Department of Obstetrics and Gynecology, Peking Union Medical College Hospital, Chinese Academy of Medical Sciences & Peking Union Medical College, Beijing, China; 2 Department of Pathology, Peking Union Medical College Hospital, Chinese Academy of Medical Sciences & Peking Union Medical College, Beijing, China; Zhejiang University School of Medicine, CHINA

## Abstract

**Objectives:**

To compare the clinicopathologic features and prognosis of Chinese patients with ovarian clear cell carcinoma (CCC) and serous carcinoma (SC).

**Methods:**

A retrospective cohort study was designed to investigate the clinicopathologic characteristic and prognosis of patients with CCC and SC who were diagnosed and treated in in a tertiary referral center (Peking Union Medical College Hospital) between 1999 and 2009. The Kaplan-Meier method and Cox regression were employed in the survival analysis.

**Results:**

A total of 504 cases were included in the study, comprising 197 cases of CCC and 307 cases of SC. The mean age of the patients with SC was greater than of CCC patients (3.6±0.94, *P*<0.001). Patients with CCC were more likely to be early-stage and optimally debulked (*P*<0.001). Regarding cancer-antigen 125, 22% of the patients with CCC had normal values, and the level was significantly lower than in patients with SC (*P*<0.001). More CCC patients had platinum-resistant tumors compared with platinum-sensitive disease (45.7% in CCC vs. 61.0% in SC [*P*=0.008]). The 5-year survival rate was 51.2% in the CCC group vs. 49.8% in the SC group (*P*=0.428). Patients with advanced CCC had a statistically significant poorer overall survival (OS) compared with their SC counterparts (38.0 vs. 52.0 months; hazard ratio 1.584, 95% confidence interval [CI] 1.167-2.150, *P*=0.003). However, the advantage of improved progression-free survival (PFS) existed across all stages.

**Conclusions:**

Women with ovarian CCC presented at a younger age and early stage. Patients with ovarian CCC also had improved PFS, but they had similar OS compared to patients with SC. However, patients with advanced CCC had decreased survival.

## Introduction

Epithelial ovarian carcinoma (EOC) is the most lethal gynecologic malignancy [1]. The histological subtypes of ovarian cancer are perceived to be distinct diseases, each with distinct clinical and molecular characteristics [2–4]. In addition, a recently published systematic review demonstrated that the relative frequencies of the histological subtypes were not homogenous across countries [5]. In general, ovarian clear cell carcinoma (CCC) is the second-most common type of EOC, accounting for 5–25% of cases depending on geographic locations [4], with a median frequency of 5.3% [5]in the published literature.

Historically, the diagnosis of ovarian CCC has been of great concern due to the fact that CCC has been reported to confer a poorer prognosis than the more common serous carcinoma (SC) [6]. However, published studies comparing ovarian CCC with other EOC subtypes have shown discrepancies due to the rarity of such cases [7, 8]. With accumulating case outcomes, there have been several important publications with large sample sizes based on database analyses and clinical trials regarding the comparative prognosis of CCC vs. SC [8–15]. Nevertheless no definite agreement has ever been achieved. In addition, most of the above-mentioned studies came from European countries [9, 10, 12–14, 16], one from the United States based on SEER (Surveillance, Epidemiology, and End Results) [11] and the other cohort study from Japan [15]. Few studies concerning ovarian CCC prognosis in Chinese patients have ever been reported. The purpose of the current comparative study was to analyze and compare the clinicopathologic characteristic and prognosis of patients with CCC and SC based on Chinese patients’ data, which we hope might bring new information to the clinical management of ovarian CCC.

## Materials and Methods

### 1. Study patients

Approved by the Institutional Review Boards and Ethical Committee, this retrospective cohort study was conducted at a tertiary referral center (Peking Union Medical College Hospital). All of the patients gave their written informed consent prior to inclusion in the study. We searched the Electronic Medical Records (EMR) Database in our institution to identify all of the patients with EOC diagnosed and treated between December 1999 and December 2009. Cases were selected mainly based on the original diagnosis by the pathologists via the pathology results available in EMR. In our previous work (unpublished), a SC database (selecting cases by random-number) and tissue microarray was constructed. In the current series, we included all of the patients with a diagnosis of ovarian CCC in the same period to set up a CCC database due to the rarity of such cases. In the process, a comprehensive retrospective review of the medical charts was performed by two different well-trained gynecologic doctors; the same senior gynecologic oncologist (Dr. Jiaxin Yang) was responsible for quality-control by checking the extraction results. The inclusion criteria were pathologically confirmed CCC or SC of the ovary, available medical records for pretreatment assessment, diagnosed and/or treated in our institution and complete follow-up information. The exclusion criteria were listed as follows: multiple medical complications (at least two comorbidities), mixed epithelial histology and incomplete follow-up information.

Patients’ demographics, clinical features, treatments and survival information were abstracted from the EMR. The variables assessed were as follows: age at the time of diagnosis, serum level of cancer-antigen 125 (CA-125) before treatment, date and type of primary surgery, International Federation of Gynecology and Obstetrics (FIGO) stage, residual disease, lymph node metastasis, adjuvant chemotherapy, time of disease progression or recurrence and survival status at last contact.

Microscopic slides were reviewed by the same gynecology-dedicated pathologist (Dr. Yan You) and confirmed by a second experienced gynecologic pathologist (Dr. Jie Chen). Both were blinded to the original diagnosis. The histologic subtype was classified according to the World Health Organization (WHO) definitions [17]. Our previous study suggested that mixed-type CCC patients had a poorer survival than their pure CCC counterparts, though statistical significance was not reached [18]. To ensure purity, cases with mixed histology were excluded from the current study. Given that most of the aforementioned publications did not distinguish ovarian low-grade and high-grade serous carcinoma, we also compared the CCC cases to the aggregated SC types as a whole. Ovarian clear-cell tumors were not graded because one of the main difficulties in managing CCC is the histologic grading [12, 19].

Patients were staged using the FIGO 2012 staging system and further divided into early stage (FIGO I-II) and advanced stage (FIGO III-IV) for the purpose of statistical analysis. In our institution, complete staging surgery and cytoreductive surgery (CRS) with subsequent adjuvant chemotherapy is considered the standard approach for ovarian cancer patients with early-stage and advanced-stage disease, respectively. The vast majority of the patients received platinum-based chemotherapy regimens with the number of cycles ranging from six to nine. Operations were performed by gynecologic oncology faculty members to achieve optimal cytoreduction, which was defined as residual disease less than (or including) 1 cm after primary debulking. The platinum-sensitive disease group included patients who had relapsed more than six months after completion of the last platinum-based regimen. CA-125 was categorized as normal (≤ 35 U/ml) or high (> 35 U/ml), consistent with the definition of CA-125 normalization commonly used in the clinical setting.

The duration of the patients’ overall survival (OS) was calculated from the date of their primary surgery to the date of death or last contact, and their progression-free survival (PFS) was measured from the date of their primary surgery to the date of first progression or recurrence. All of the follow-up information was censored following December 31, 2012.

### 2. Statistical analyses

Continuous variables were evaluated by parametric Student *t* tests or Mann-Whitney *U* tests as appropriate, while categorical variables were compared by chi-squared tests. Probability of superiority/probabilistic index = Mann-Whitney U value/mn (number of participants) [20]. PFS and OS times were estimated using Kaplan-Meier model (Mantel-Cox test) while Cox regression was employed for multivariate analysis. Variables with statistical significance in univariate analysis were included in the multivariate one. All of the *P* values reported were two-sided, and a value of *P*<0.05 was considered statistically significant. Statistical Package for Social Science (SPSS) statistical software (Version 17.0, SPSS, Inc., Chicago, IL, USA) and GraphPad Prism (Version 5.0, GraphPad Software, Inc., La Jolla, CA, USA) were used for all of the analyses.

## Results

The preliminary EMR database search identified 1543 cases of EOC during the 10-year period 1999–2009. A rough estimation of the histological subtype distribution was as follows: SC, 875 cases (56.7%); endometrioid carcinoma, 239 cases (15.5%); CCC, 202 cases (13.1%); mucinous carcinoma, 73 cases (4.7%) and the other 154 cases (10.0%) including mixed epithelial carcinoma, transitional cell tumor and undifferentiated carcinoma. Then, two gynecologic doctors conducted a comprehensive review of the medical charts for the 202 cases with CCC and a randomly-chosen subset of 322 cases with SC. A total of 20 cases (5 in the CCC group and 15 in the SC group) were excluded due to incomplete follow-up information. Therefore, the current comparative study was based on 197 patients with CCC and 307 patients with SC.

### 1. Comparison of clinical and pathological features

Table 1 shows the clinical and pathological characteristics of the entire cohort. The mean age of patients with a diagnosis of clear cell histology was 50 years with a range of 29 to 81 years. They were significantly younger than the patients with a diagnosis of serous histology (Difference ± Standard Error 3.6±0.94, 95% confidence interval [CI] 1.7–5.4; *P*<0.001). Women with CCC were more likely to present with early-stage disease compared with SC patients (48.7% for CCC and 16.3% for SC [*P*<0.001]).

**Table 1:** Clinicopathological features of patients with clear cell carcinoma and serous carcinoma.

Characteristics	CCC (n = 197)	SC (n = 307)	*P* value
Mean age (range) (years)	50 (29–81)	54 (22–83)	<0.001^a^
FIGO stage			
Stage I	83 (42.1%)	20 (6.5%)	<0.001^b^
Stage II	13 (6.6%)	30 (9.8%)	
Stage III	93 (47.2%)	237 (77.2%)	
Stage IV	8 (4.1%)	20 (6.5%)	
Stage Group			
Early (I+II)	96 (48.7%)	50 (16.3%)	<0.001^b^
Advanced (III+IV)	101 (51.3%)	257 (83.7%)	
Pre-operation CA-125 (the 50th Percentile [min-max]) (U/ml)	101.0 (3.1–14647.0)	870.0 (8.0–56541.0)	<0.001^c^
	n = 173	n = 300	
Pre-chemotherapy CA-125 (the 50th Percentile [min-max]) (U/ml)	75.0 (0.1–5000)	147.9 (4.8–9940)	<0.001^c^
	n = 160	n = 293	
Residual disease			
≤1 cm (optimal)	121 (61.4%)	129 (42.0%)	<0.001^b^
>1 cm (suboptimal)	76 (38.5%)	178 (58.0%)	
Lymph node metastasis	43 (25.1%)	128 (49.4%)	<0.001^b^
	n = 171	n = 259	
Adjuvant chemotherapy	192 (97.5%)	306 (99.7%)	0.070^b^

^a^ Student’s t test

^b^ Chi-squared test

^c^ Mann-Whitney U test

Abbreviations: CCC, clear cell carcinoma; SC, serous carcinoma; CA-125, cancer-antigen 125; CI, confidence interval; min, minimum; max, maximum.

Serum CA-125 levels were reported before the primary surgery in 473 patients (173 with CCC and 300 with SC). The baseline CA-125 level was significantly lower among the CCC patients (probabilistic index = 0.272, *P*<0.001, Mann-Whitney *U* test). Normal CA-125 levels were observed in 38 (22.0%) patients with CCC, whereas only 2.3% of patients with SC had normal levels (*P*<0.001, Chi-squared test). Pre-chemotherapy CA-125 levels (measured following surgical debulking) were available for 160 patients with CCC and 293 patients with SC. Interestingly, the SC patients experienced a steep reduction in CA-125 level following their primary surgery from 870.0 U/ml to 147.9 U/ml. At that time, the CA-125 levels was still significantly different between the two groups (probabilistic index = 0.359, *P*<0.001, Mann-Whitney *U* test).

At the completion of the primary surgery, 61.4% of the patients in the CCC group and 42.0% of patients in the SC group had residual disease ≤1 cm (*P*<0.001), in which the odds ration was 2.20. Of the entire study population, 85.3% (430/504) underwent lymphadenectomy. Among them, 25.1% (43/171) of patients in the CCC group and 49.4% (128/259) of patients in the SC group were found to have lymph node metastasis, and this difference was statistically significant (*P*<0.001). The vast majority (98.8%) of the patients received adjuvant chemotherapy, and almost all of the patients received platinum-based regimens.

### 2. Comparison of prognosis and survival outcomes

The mean follow-up was 51 months (range 1–152 months) for the entire cohort. During the study period, 70% (360/507) of the patients developed disease progression and/or relapse, including 51.3% (101/197) of the patients in the CCC group and 84.4% (259/307) of the patients in the SC group. Among them, 50 patients (14 in the CCC and 36 in the SC group) had progressive disease during the first-line chemotherapy, *P* = 0.090). In terms of response to platinum-based chemotherapy, 45.6% (46/101) of the CCC patients and 61.0% (158/259) of the SC patients had platinum-sensitive disease, and this difference was statistically significant (*P* = 0.008). During the study period, 97(97/197, 49.2%) and 168(168/307, 54.7%) deaths were observed in the CCC and SC group respectively, leading to a total death of 265.

**Fig 1:**
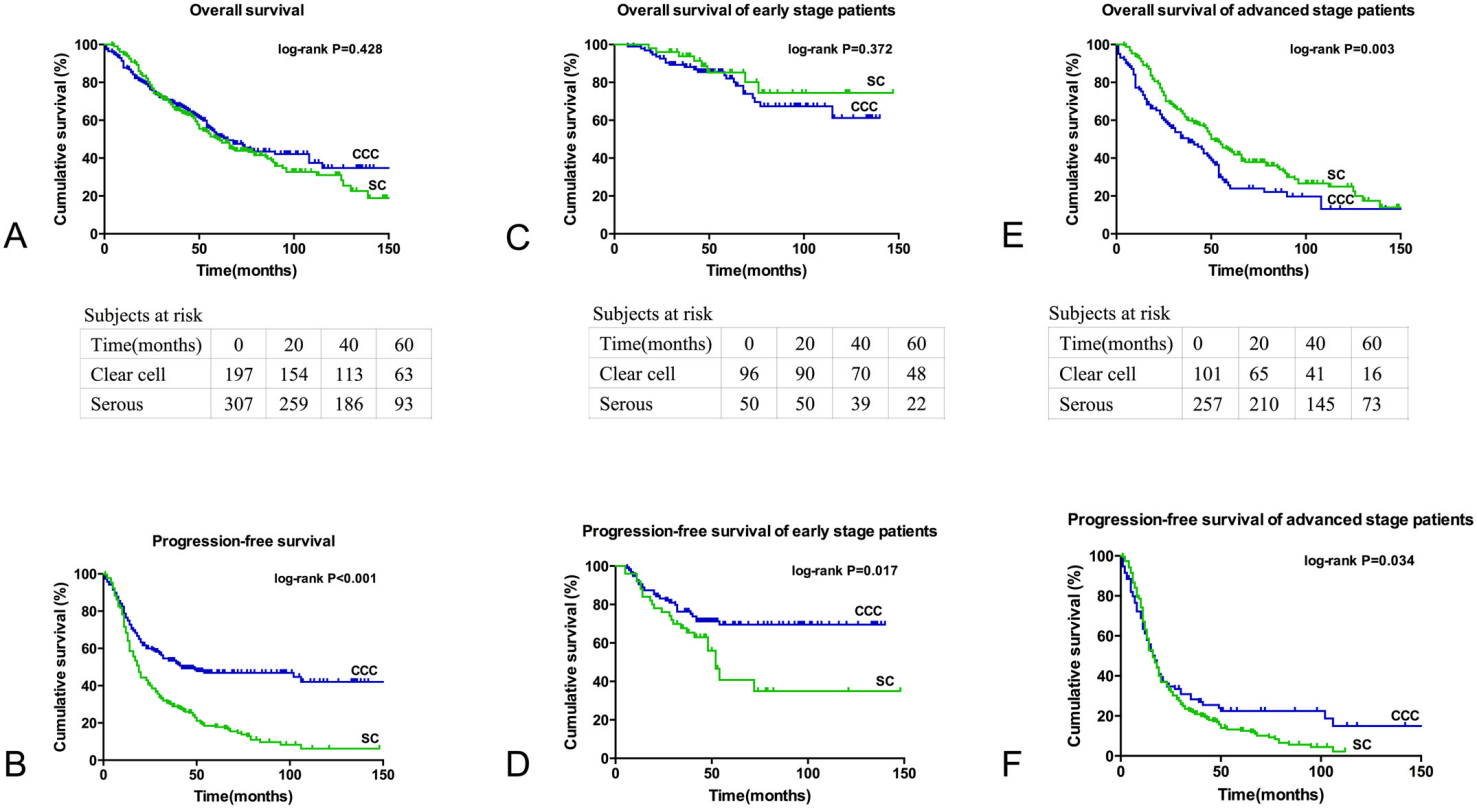
Kaplan-Meier survival curves of patients with ovarian clear cell carcinoma (CCC) and serous carcinoma (SC). Fig 1A and 1B show the OS and PFS of CCC and SC, respectively. Fig 1C and 1D show the survival curves of patients with early-stage disease, and Fig 1E and 1F show the survival curves of patients with late-stage disease.

The OS for the CCC and SC group was not significantly different (Fig 1A; *P* = 0.428). On the whole, patients had an estimated median OS of 64.0 months (95% CI 48.7–79.3 months) in the CCC group and 60.0 months (95% CI 49.9–70.1 months) in the SC group with a hazard ratio (HR) of 0.901 (95% CI 0.703–1.153). From the survival table based on the Kaplan-Meier method, the 5-year survival rates were 51.2% and 49.8% in patients with CCC and SC, respectively. In contrast, improved PFS was observed in the CCC group (Fig 1B; *P*<0.001). These patients had a median PFS of 41.0 months (95% CI 0.0–86.2 months) in the CCC group compared with 16.0 months (95% CI 13.8–18.2 months) in the SC group.

Table 2 shows the survival outcome matching for age at diagnosis, stage of disease and debulking status. After adjusting for age, CCC patients had a better PFS than SC patients with no significant difference in OS. In early-stage disease, SC patients had a slightly better 5-year OS compared with their CCC counterparts (85.2% vs. 82.0%) without statistical significance (*P* = 0.372). However, a better PFS was identified in the CCC group (not achieved in the study period vs. 52.0 months [*P* = 0.017]). In regard to advanced disease, patients with CCC had a poorer OS with a median survival of 38.0 months (95% CI 23.7–52.3 months) compared with 52.0 months (95% CI 45.7–58.3 months) in the SC group (hazard ratio 1.584, 95% CI 1.167–2.150, *P* = 0.003). The PFS advantage still existed among patients with late-stage disease, though with only a minor difference (16.0 months, 95% CI 12.2–19.8 months for CCC and 14.0 months, 95% CI 12.0–16.0 months for SC [HR 1.318, 95% CI 1.013–1.715; *P* = 0.034]).

**Table 2:** Survival outcome matching for age, stage of disease and debulking status.

	Overall survival (Median/95%CI) (Months)			Progression-free survival (Median/95%CI) (Months)		
	CCC	SC	P value	CCC	SC	P value
Age						
Age≤53*	115	59	0.132	86	18	<0.001
	50.4–179.5	40.4–77.4		69.7–102.5	14.7–21.2	
Age>53	54	61	0.672	30	14	<0.001
	41.8–66.2	50.8–71.2		5.5–54.5	11.3–16.7	
Stage						
Early (I+II)	NA#	NA	0.372	NA	52	0.017
					45.1–58.9	
Late (III+IV)	38	52	0.003	16	14	0.034
	23.7–52.3	45.7–58.3		12.2–19.8	12.0–16.0	
Debulking						
Optimal	NA	76	<0.001	NA	23	<0.001
		55.4–96.6			16.8–29.1	
Suboptimal	27	50	<0.001	12	12	0.906
	18.0–35.9	37.6–62.4		8.7–15.3	10.4–13.6	

*The mean age of the entire cohort was 53 years old.

^#^NA (not achieved): at time of analysis, median survival had not been reached.

Abbreviations: CCC, clear cell carcinoma; SC, serous carcinoma; CI, confidence interval; NA, not achieved.

Table 3 presents univariate and multivariate survival analysis. Late stage (*P*<0.001), and suboptimal debulking (*P*<0.001) were independent negative predictors for OS. On the other hand, late stage (*P*<0.001), suboptimal debulking (*P*<0.001), and serous histology (*P* = 0.005) were statistically significant adverse variable for PFS.

**Table 3:** Significant predictors of survival in univariate (Kaplan-Meier method using log-rank test) and multivariate survival analysis (Cox regression).

Univariate						
Variable	Category	No.	OS		PFS	
		(Percentage)	Median	P	Median	P
Age (years)	≤53	265(52.6%)	66	0.211	23	0.066
	>53	239(47.4%)	56		18	
Stage	Early	146(29.0%)	NA	**<0.001**	NA	**<0.001**
	Late	358(71.0%)	49		14	
Debulking	Optimal	250(49.6%)	96	**<0.001**	42	**<0.001**
	Suboptimal	254(50.4%)	41		12	
Lymph node metastasis	Yes	170(39.6%)	49	**<0.001**	14	**<0.001**
	No	259(60.4%)	108		41	
Histology	Clear cell	197(39.1%)	64	0.428	41	**<0.001**
	Serous	307(60.9%)	60		16	
Multivariate						
Variable	OS			PFS		
	HR	95% CI	*P*	HR	95% CI	*P*
Early stage	2.896	1.846–4.544	**<0.001**	2.536	1.745–3.687	**<0.001**
Optimal debulking	1.917	1.396–2.633	**<0.001**	1.961	1.492–2.579	**<0.001**
Lymph node metastasis	0.746	0.551–1.012	0.060	0.895	0.690–1.161	0.403
Clear cell carcinoma	—	—	—	1.482	1.125–1.953	**0.005**

Abbreviations: OS, overall survival; PFS, progression-free survival; NA, not achieved; HR, Hazard Ratio; CI, confidence interval.

## Discussion

Sung and colleagues undertook a systematic review including 17 database analyses, six clinical trials, four cohort studies and 14 molecular studies of surgical sample archives to investigate the global distribution pattern of the histological subtypes of EOC [5]. They arrived at a conclusion that EOC is a heterogeneous disease with a heterogeneous distribution pattern. Globally, the median relative frequencies were as follows: serous, 45.0%; endometrial, 12.6%; and clear cell, 5.3%. The present study focused on patients with EOC in mainland China and identified the histology distribution pattern from a tertiary referral center. Our results revealed that serous carcinoma was the leading subtype, accounting for 56.7% of cases, followed by the endometrioid (15.5%) and clear cell (13.1%) subtypes. Our results suggested that SC was the most commonly reported subtype of EOC in China but that the CCC proportion was highly elevated compared with the global mean value.

This study revealed that patients with ovarian CCC tended to be diagnosed at a younger age, at an earlier FIGO stage and with a much lower serum CA-125 level before treatment. We were interested in CA-125 because the serum level of CA-125 is well established as a highly useful marker for monitoring treatment response [19, 21]. Nevertheless the role and prognostic implication of CA-125 for CCC specifically is less certain [19]. In our series, approximately one-fifth of the ovarian CCC patients had normal CA-125 levels at presentation. In addition, the baseline CA-125 level prior to surgery in the CCC patients was lower than that in SC patients. It is worth noting that the CCC patients only had a minor reduction in their CA-125 levels compared with SC patients, though both patient groups were optimally debulked. Tian and colleagues reviewed seven Gynecologic Oncology Group phase III trials to illuminate the relationship between CA-125 and prognosis in advanced clear cell and mucinous cancers [19]. They found that the pre-chemotherapy CA-125 level in CCC was approximately one-half compared with other EOC histologies. They postulated that this result might be due to the smaller volume of residual disease as well as fundamental differences in the biology of malignancies. Even within the same histology subtype, the wide distribution of preoperative CA-125 might be due to the differences of tumor stage, histologic grade and the presence of ascites [22].

Ovarian CCC patients were more likely to have early-stage disease. A British Columbia study reviewed 39.5% of 2555 cases registered in a population-based EOC database and found that CCC accounted for 24.7% of cases of early-stage tumors (FIGO I+II) on a background of only 9.5% of patients having histologically proven CCC [23]. Consistent with the higher early-stage frequency, more tumors in the CCC group were surgically resectable, though with different rates of optimal cytoreduction. Our study suggested that Chinese patients with ovarian CCC were more likely to present with early-stage disease compared with SC (48.7% for CCC and 16.3% for SC [*P*<0.001]). Optimal debulking was achieved in 61.4% of the ovarian CCC patients, compared to 42.0% of the patients with SC (*P*<0.001). An even higher proportion of SC patients had positive lymph node metastases (49.4% vs. 25.1% [*P*<0.001]). However, with respect to adjuvant chemotherapy, the CCC patients were more resistant to platinum-based regimens (platinum-sensitive disease in 45.6% of CCC cases vs. 61.0% of SC cases [*P* = 0.008]). Resistance to chemotherapy might be an important impact factor for the survival in patients with ovarian CCC.

Across all stages, patients with CCC had a better PFS outcome than patients with SC. However, no significant difference was found in OS between these two groups. Further subgroup analysis demonstrated that patients with late-stage CCC had worse OS than patients with late-stage SC (38.0 months vs. 52.0 months, respectively [*P* = 0.003]). Despite the great efforts made to explore the relative survival rates of ovarian CCC patients compared SC patients, no clear consensus has yet been reached to understand these differences. A large study based on the SEER database consisting of 1411 cases of ovarian CCC found that stage I CCC had a similar OS to SC (85.3% vs. 86.4%) [11]. This finding was also supported by a large European randomized trial on early-stage (FIGO I+II) patients with 5-year survival rates of 71% and 61% for CCC and SC, respectively [12]. A conflicting result was reported in a British Columbia study, which reported that the 10-year OS was 87% for 35 patients with stage Ia/Ib CCC compared with 68% for patients with high-grade SC [13]. The results have also varied for different studies on patients with advanced diseases. A Japanese cohort study including 178 cases of CCC and 311 cases of SC demonstrated that patients with stage IIIb/IIIc CCC had a significantly worse prognosis than patients with SC [15]. Mackay and colleagues analyzed seven randomized trials to conclude that the median OS for CCC and SC were 21.3 and 40.8 months, respectively [14]. That study also demonstrated that patients with CCC had shorter times to progression (HR 1.6, 95% CI 1.4–1.9) [14]. In the current study, CCC patients had similar OS compared to SC patients regardless of stage. However, decreased survival of CCC patients was observed in advanced disease (median OS 38.0 vs. 52.0 months [HR 1.584, 95% CI 1.167–2.150; *P* = 0.003]). Regarding PFS, patients with ovarian CCC had an advantage across all stages.

When interpreting the data of this study, several potential points must be noted. First, the cohort is limited by the selection and surveillance biases often associated with studies from single academic institutions. A nationwide, collaborative study is needed to truly present the clinical profile of Chinese patients with ovarian CCC. Second, the lack of data on post-recurrent management is another major limitation of our study. Specific explanation and evaluation of the treatment after relapse would make the comparison of survival outcomes more reliable. Furthermore, the distinction between low-grade and high-grade ovarian SC [24], which are regarded as distinct entities, was not taken into consideration in the present study to increase comparability with previous published works [8]. A more accurate and representative frequency pattern would be available by further investigation.

## Conclusion

Our study illustrated that ovarian CCC and SC accounted for 13.1% and 56.7% of all EOC cases, respectively. Women with ovarian CCC usually presented at a younger age and earlier stage. They had similar OS to SC patients on the whole, though patients with advanced CCC had decreased survival. In terms of PFS, patients with ovarian CCC had an advantage across all stages.

## References

[1] Siegel R, Ma J, Zou Z, Jemal A. Cancer statistics, 2014. CA Cancer J Clin. 2014;64(1):9–29. 10.3322/caac.21208 24399786

[2] del Carmen MG, Birrer M, Schorge JO. Clear cell carcinoma of the ovary: a review of the literature. Gynecol Oncol. 2012;126(3):481–90. 10.1016/j.ygyno.2012.04.021 22525820

[3] Kobel M, Kalloger SE, Boyd N, McKinney S, Mehl E, Palmer C, et al Ovarian carcinoma subtypes are different diseases: implications for biomarker studies. PLoS Med. 2008;5(12):e232 10.1371/journal.pmed.0050232 19053170 PMC2592352

[4] Okamoto A, Glasspool RM, Mabuchi S, Matsumura N, Nomura H, Itamochi H, et al Gynecologic Cancer InterGroup (GCIG) consensus review for clear cell carcinoma of the ovary. Int J Gynecol Cancer. 2014;24(9 Suppl 3):S20–5. 10.1097/IGC.0000000000000289 25341576

[5] Sung PL, Chang YH, Chao KC, Chuang CM. Global distribution pattern of histological subtypes of epithelial ovarian cancer: a database analysis and systematic review. Gynecol Oncol. 2014;133(2):147–54. 10.1016/j.ygyno.2014.02.016 24556058

[6] Goff BA, Sainz de la Cuesta R, Muntz HG, Fleischhacker D, Ek M, Rice LW, et al Clear cell carcinoma of the ovary: a distinct histologic type with poor prognosis and resistance to platinum-based chemotherapy in stage III disease. Gynecol Oncol. 1996;60(3):412–7. 8774649 10.1006/gyno.1996.0065

[7] Tan DS, Kaye S. Ovarian clear cell adenocarcinoma: a continuing enigma. J Clin Pathol. 2007;60(4):355–60. 17018684 10.1136/jcp.2006.040030 PMC2001101

[8] Lee YY, Kim TJ, Kim MJ, Kim HJ, Song T, Kim MK, et al Prognosis of ovarian clear cell carcinoma compared to other histological subtypes: a meta-analysis. Gynecol Oncol. 2011;122(3):541–7. 10.1016/j.ygyno.2011.05.009 21640372

[9] Trimbos JB, Parmar M, Vergote I, Guthrie D, Bolis G, Colombo N, et al International Collaborative Ovarian Neoplasm trial 1 and Adjuvant ChemoTherapy In Ovarian Neoplasm trial: two parallel randomized phase III trials of adjuvant chemotherapy in patients with early-stage ovarian carcinoma. J Natl Cancer Inst. 2003;95(2):105–12. 12529343

[10] Pectasides D, Fountzilas G, Aravantinos G, Kalofonos C, Efstathiou H, Farmakis D, et al Advanced stage clear-cell epithelial ovarian cancer: the Hellenic Cooperative Oncology Group experience. Gynecol Oncol. 2006;102(2):285–91. 16516283 10.1016/j.ygyno.2005.12.038

[11] Chan JK, Teoh D, Hu JM, Shin JY, Osann K, Kapp DS. Do clear cell ovarian carcinomas have poorer prognosis compared to other epithelial cell types? A study of 1411 clear cell ovarian cancers. Gynecol Oncol. 2008;109(3):370–6. 10.1016/j.ygyno.2008.02.006 18395777

[12] Timmers PJ, Zwinderman AH, Teodorovic I, Vergote I, Trimbos JB. Clear cell carcinoma compared to serous carcinoma in early ovarian cancer: same prognosis in a large randomized trial. Int J Gynecol Cancer. 2009;19(1):88–93. 10.1111/IGC.0b013e3181991546 19258948

[13] Kobel M, Kalloger SE, Santos JL, Huntsman DG, Gilks CB, Swenerton KD. Tumor type and substage predict survival in stage I and II ovarian carcinoma: insights and implications. Gynecol Oncol. 2010;116(1):50–6. 10.1016/j.ygyno.2009.09.029 19822358

[14] Mackay HJ, Brady MF, Oza AM, Reuss A, Pujade-Lauraine E, Swart AM, et al Prognostic relevance of uncommon ovarian histology in women with stage III/IV epithelial ovarian cancer. Int J Gynecol Cancer. 2010;20(6):945–52. 10.1111/IGC.0b013e3181dd0110 20683400

[15] Mizuno M, Kikkawa F, Shibata K, Kajiyama H, Ino K, Kawai M, et al Long-term follow-up and prognostic factor analysis in clear cell adenocarcinoma of the ovary. J Surg Oncol. 2006;94(2):138–43. 16847906 10.1002/jso.20251

[16] Vergote I, De Brabanter J, Fyles A, Bertelsen K, Einhorn N, Sevelda P, et al Prognostic importance of degree of differentiation and cyst rupture in stage I invasive epithelial ovarian carcinoma. Lancet. 2001;357(9251):176–82. 11213094 10.1016/S0140-6736(00)03590-X

[17] Tavassoli FA, Devilee P. Pathology and genetics of tumours of the breast and female genital organs: Iarc; 2003.

[18] Ye S, You Y, Yang J, Cao D, Bai H, Huang H, et al Comparison of pure and mixed-type clear cell carcinoma of the ovary: a clinicopathological analysis of 341 chinese patients. Int J Gynecol Cancer. 2014;24(9):1590–6. 10.1097/IGC.0000000000000275 25254564

[19] Tian C, Markman M, Zaino R, Ozols RF, McGuire WP, Muggia FM, et al CA-125 change after chemotherapy in prediction of treatment outcome among advanced mucinous and clear cell epithelial ovarian cancers: a Gynecologic Oncology Group study. Cancer. 2009;115(7):1395–403. 10.1002/cncr.24152 19195045 PMC2743569

[20] Erceg-Hurn DM, Mirosevich VM. Modern robust statistical methods: an easy way to maximize the accuracy and power of your research. Am Psychol. 2008;63(7):591–601. 10.1037/0003-066X.63.7.591 18855490

[21] Rustin GJ, Marples M, Nelstrop AE, Mahmoudi M, Meyer T. Use of CA-125 to define progression of ovarian cancer in patients with persistently elevated levels. J Clin Oncol. 2001;19(20):4054–7. 11600607 10.1200/JCO.2001.19.20.4054

[22] Cooper BC, Sood AK, Davis CS, Ritchie JM, Sorosky JI, Anderson B, et al Preoperative CA 125 levels: an independent prognostic factor for epithelial ovarian cancer. Obstet Gynecol. 2002;100(1):59–64. 12100804 10.1016/s0029-7844(02)02057-4

[23] Kobel M, Kalloger SE, Huntsman DG, Santos JL, Swenerton KD, Seidman JD, et al Differences in tumor type in low-stage versus high-stage ovarian carcinomas. Int J Gynecol Pathol. 2010;29(3):203–11. 10.1097/PGP.0b013e3181c042b6 20407318

[24] Vang R, Shih Ie M, Kurman RJ. Ovarian low-grade and high-grade serous carcinoma: pathogenesis, clinicopathologic and molecular biologic features, and diagnostic problems. Adv Anat Pathol. 2009;16(5):267–82. 10.1097/PAP.0b013e3181b4fffa 19700937 PMC2745605

